# Muscle density, but not size, is independently associated with cognitive health in older adults with hip fractures

**DOI:** 10.1093/jbmrpl/ziae047

**Published:** 2024-04-02

**Authors:** Yufeng Ge, Qian You, Feng Gao, Gang Liu, Ling Wang, Bo Li, Maoyi Tian, Minghui Yang, Xinbao Wu

**Affiliations:** Department of Orthopaedics and Traumatology, Beijing Jishuitan Hospital, Capital Medical University, Beijing 100035, China; Department of Neurology, Beijing Jishuitan Hospital, Capital Medical University, Beijing 100035, China; Department of Orthopaedics and Traumatology, Beijing Jishuitan Hospital, Capital Medical University, Beijing 100035, China; Department of Orthopaedics and Traumatology, Beijing Jishuitan Hospital, Capital Medical University, Beijing 100035, China; Department of Radiology, Beijing Jishuitan Hospital, Capital Medical University, Beijing 100035, China; JST Sarcopenia Research Center, Beijing Research Institute of Traumatology and Orthopaedics, Beijing 100035, China; Department of Orthopaedics and Traumatology, Beijing Jishuitan Hospital, Capital Medical University, Beijing 100035, China; The George Institute for Global Health, Peking University Health Science Centre, Beijing 100191, China; Department of Orthopaedics and Traumatology, Beijing Jishuitan Hospital, Capital Medical University, Beijing 100035, China; Department of Orthopaedics and Traumatology, Beijing Jishuitan Hospital, Capital Medical University, Beijing 100035, China

**Keywords:** muscle density, muscle size, cognitive impairment, hip fractures, computed tomography

## Abstract

Emerging evidence indicates a complex interplay between skeletal muscle and cognitive function. Despite the known differences between muscle quantity and quality, which can be measured via computed tomography (CT), the precise nature of their associations with cognitive performance remain underexplored. To investigate the links between muscle size and density and cognitive impairment (CI) in the older adults with hip fractures, we conducted a post hoc, cross-sectional analysis within a prospective cohort study on 679 patients with hip fractures over 65. Mini-Mental State Examination (MMSE) and routine hip CT imaging were utilized to assess cognition function and muscle characteristics in older adults with hip fractures. The CT scans provided data on cross-sectional area and attenuation for the gluteus maximus (G.MaxM) and the combined gluteus medius and minimus (G.Med/MinM). Participants were categorized into CI and non-CI groups based on education levels and MMSE scores. Multivariate logistic regressions, propensity score (PS) methods, and subgroup analysis were employed to analyze associations and validate findings. This study included 123 participants (81.6 ± 6.8 years, 74% female) with CI and 556 participants (78.5 ± 7.7 years, 72% female) without. Compared to the non-CI group, muscle parameters, especially density, were significantly lower in the CI group. Specifically, G.Med/Min muscle density, but not size was robustly associated with CI (odds ratio (OR) = 0.77, 95% confidence interval = 0.62–0.96, *P* = 0.02), independent of other medical situations. Sensitivity analysis corroborated that G.Med/Min muscle density was consistently lower in the CI group than the non-CI group, as evidenced in the PS matched (*P* = 0.024) and weighted cohort (*P* = 0.033). Enhanced muscle parameters, particularly muscle density in the G.Med/MinM muscle, correlate with a lower risk of CI. Muscle density demonstrates a stronger association with cognitive performance than muscle size, highlighting its potential as a key focus in future cognitive health research.

## Graphical Abstract

**Graphical Abstract:**
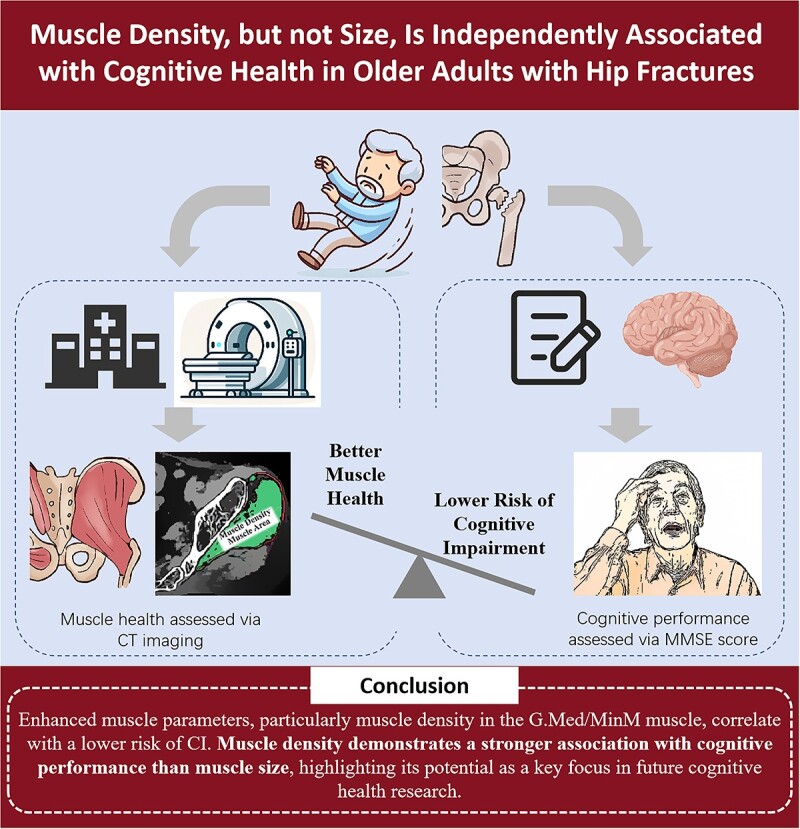

## Introduction

The aging population is a pressing global issue. Degeneration of the brain and muscles occurs rapidly in the sixth decade of life and throughout the remaining lifespan, resulting in cognitive impairment (CI) and muscle function decline.^1^^,^^2^ In China, a national cross-sectional study revealed that 15.5% of adults aged 60 years and above (~38.8 million individuals) experience mild CI,^3^ while sarcopenia affects about 17.4% of community-dwelling older adults over 65.^4^ Recent years have seen growing interest in the shared inflammatory and hormonal pathways between these conditions, indicating potential brain–muscle interactions.^5–8^

Hip fractures, common and serious injuries among the older adults, are notably linked with CI and sarcopenia. More than 30% of the older population with hip fractures suffer from either condition.^9^^,^^10^ Despite its significance, the relationship between cognitive function and muscle health has seen limited exploration, partly due to difficulties in assessing muscle attributes in patients with hip fractures. However, computed tomography (CT), commonly used in evaluating hip fracture, offers a valuable opportunity for precise body composition analysis in the older adults.^11^ Computed tomography scans of the hip provide intricate details on muscle composition, highlighting both the volume and quality.^12^ Previous studies utilizing CT have pinpointed the density of the gluteus medius and minimus (G.Med/Min) muscles as a significant predictor for both initial and subsequent hip fractures,^13^^,^^14^ yet the connection between CT-visible muscle characteristics and CI has not been thoroughly investigated.

In this post-hoc analysis, we utilized baseline data from a prospective cohort study, leveraging routine CT scans in the clinical management of hip fractures to obtain muscle measurements. Our objectives were twofold: to investigate the association between hip muscle size and density with cognitive performance in the older adults with hip fractures, and to determine if these muscle parameters independently correlate with cognition. We hypothesized that muscle density has a stronger association with CI than muscle size.

## Materials and methods

### Study design

We performed a post-hoc analysis of our previous prospective, controlled study, which explored the effect of a co-management care model on older hip fracture patients in China.^15^ Ethics approval was obtained from the Institutional Review Board of the hospital (201807-II) before the initiation of our previous study. In this cross-sectional analysis, we investigated and compared CT-based muscle parameters with CI evaluated with the Chinese version of the Mini-Mental State Examination (MMSE).

### Participants

Between November 2018 and November 2019, 1092 patients aged 65 and older, diagnosed with hip fractures at our hospital in Beijing, China, were screened. Patients who could not walk independently before the injury or presented over 72 hours from injury were excluded to minimize changes in body composition due to bed rest after the fracture. We also ruled out those with contralateral hip lesions (like fractures or bone necrosis) that may disturb imaging measurements during screening. All participants gave written informed consent.

### CT acquisition and muscle measurements

Spiral CT imaging of the hip was conducted for all study participants upon their arrival at our emergency department before admission, utilizing two Toshiba Aquilion CT scanners (Toshiba Medical Systems Division, Tokyo, Japan). Scans were acquired in supine position from the top of the acetabulum to 3 cm below the lesser trochanter and included both legs. Scan parameters were 120 kVp, 125 mAs, 50 cm field of view, 512 × 512 matrix, 1 mm reconstructed slice thickness.

**Figure 1:**
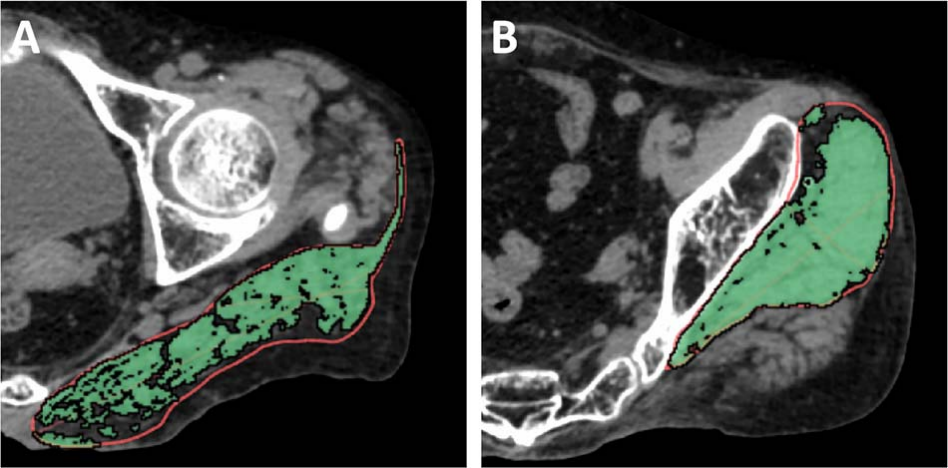
Measurement of hip muscle parameters. (A) Measurement of the gluteus maximus muscle at the level of the greater trochanter of the femur. (B) Measurement of the gluteus medius and minimus muscle at the third sacral level.

Cross-sectional area and density were measured of the G.Med/Min muscle at the level of the third sacral vertebra (S3) and of the gluteus maximus (G.Max) muscle at the level of the greater trochanter (Figure 1). All the muscle parameters were achieved from the unaffected side.

OsiriX software (Version 13.0.1, Pixmeo, Geneva, Switzerland) was used for analysis. Muscle segmentation was performed manually using the “pencil” tool to outline muscle contours. Within the resulting muscle regions of interest, a threshold of -29 HU was used to distinguish muscle tissue from fat. All muscle measurements were performed by the same investigator who had received training from an expert radiologist in CT muscle imaging prior to the analysis. The reproducibility of muscle measurement had been previously reported.^16^

### Cognitive function evaluation

Mini-Mental State Examination is an established and widely used psychometric tool for measuring cognitive performance, particularly among individuals older than 65.^17^ It is important to note, however, that the MMSE is not intended as a clinical diagnostic tool. Mini-Mental State Examination consists of a list of instructions examining six primary domains: orientation, working memory, attention, delayed memory, language, and visual construction. The scale reaches its maximum score of 30 points, with lower scores indicating poorer cognition. Notably, the cut-off point for defining CI varies according to the level of education. CI was considered if the MMSE score was less than 18 in illiterate participants, 21 in participants with elementary school education level, and 25 in participants with high-school and above education level.^18^ The Chinese version of MMSE was administered by nurses in geriatric wards through face-to-face interviews upon the participants' admission to the hospital.

### Data collection

Demographic information and perioperative records were prospectively collected. The demographic data included age, sex, weight, height, body mass index (BMI), drinking or smoking habits, education level, and living status (living alone or not, housebound (go out ≤1 time per week) or not). Baseline medical situations, such as hypertension, diabetes, heart disease, and depression, were investigated and documented from medical charts. Albumin levels were also noted. We calculated the Charlson comorbidity index (CCI)^19^ to represent the overall medical situation and further used Patient Health Questionnaire-9 (PHQ-9) to assess the affective disorders.^20^

### Statistical analysis

Data are presented as means and standard deviations for parametric data or as medians and interquartile ranges when the data are not normally distributed. Categorical variables are described using frequencies and numerical distributions. The Chi-squared test was used to assess the differences between the two groups for categorical variables and Student's t-test or the Mann–Whitney U-test for continuous variables, as appropriate (parametric vs non-parametric data, respectively).

We utilized sex-specific *Z*-score normalization to minimize sex-induced confounding bias and facilitate further analysis. Multivariate logistic regression models were used to estimate the association between muscle parameters and risk of CI, with and without age, sex, body mass index, CCI, diabetes, education, living status, housebound, albumin, and PHQ-9.

### Sensitivity analysis

The propensity score (PS) method and subgroup analysis were employed to verify the robustness of our findings. All the collected data, except the muscle parameters, were transformed into a PS for CI calculated from logistic regression. Then we established PS adjusted model, utilized PS matching^21^ and standardized mortality ratio weighting (SMRW)^22^ to enhance the precision of our study outcomes. The standardized mean difference (SMD) and a PS density plot were used to evaluate weighting efficacy in different models. Based on age (65-80 and ≥80 years), sex, BMI (<24 and ≥24 kg/m^2^), and diabetes situation, we further conducted subgroup analysis to examine potential existing interactions.

All the analyses were performed with the statistical software packages R 4.1.1 (http://www.R-project.org, The R Foundation). A two-tailed test was performed, and *P* < 0.05 was considered statistically significant.

## Results

### Population and baseline characteristics

**Figure 2:**
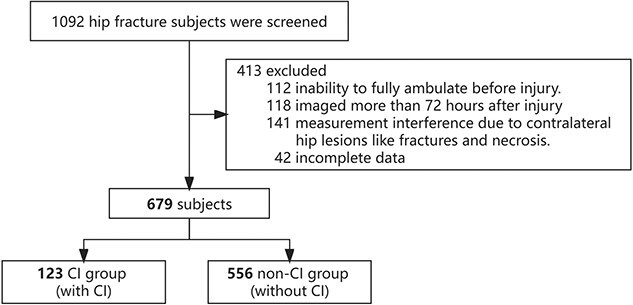
Flowchart of study. Abbreviation: CI, cognitive impairment.

A total of 679 participants were ultimately included in our final analysis, and 123 (18.1%) of them were defined as CI. Figure 2 presents the flowchart of the study.

The baseline characteristics of all available participants are listed in Table 1. The mean age was 79.04 ± 7.61 years; 489 (72%) were female. Compared with participants in the non-CI group, participants in the CI group were older (81.59 ± 6.84 vs 78.48 ± 7.66, *P* < 0.001) and more housebound (40 (32.5%) vs 86 (15.5%), *P* < 0.001) (Table 1). All muscle area and density, except G.Max muscle area, were significantly higher in the non-CI group than in the CI group (*P* < 0.05) (Table 1).

**Table 1:** Baseline characteristics.

Characteristic	Total (*n* = 679)	Non-CI (*n* = 556)	CI (*n* = 123)	*P* value
Age, years, mean ± SD	79.04 ± 7.61	78.48 ± 7.66	81.59 ± 6.84	<0.001
Female, *n* (%)	489 (72.0)	398 (71.6)	91 (74)	0.591
Weight, kg, mean ± SD	60.6 ± 11.6	60.8 ± 11.6	59.7 ± 11.9	0.378
Height, cm, mean ± SD	161.9 ± 8.1	162.0 ± 8.1	161.2 ± 7.9	0.302
BMI, kg/m^2^, mean ± SD	23.1 ± 3.7	23.1 ± 3.6	22.9 ± 4.1	0.733
Ever or current smokers, *n* (%)	116 (17.1)	97 (17.4)	19 (15.4)	0.594
Current drinkers, *n* (%)	43 (6.3)	37 (6.7)	6 (4.9)	0.464
Educational level, *n* (%)				0.088
Illiterate	118 (17.4)	101 (18.2)	17 (13.8)	
Primary school or lower	169 (24.9)	144 (25.9)	25 (20.3)	
High school	289 (42.6)	224 (40.3)	65 (52.8)	
University or higher	103 (15.2)	87 (15.6)	16 (13)	
CCI, *n* (%)				0.783
0	201 (29.6)	161 (29)	40 (32.5)	
1	241 (35.5)	200 (36)	41 (33.3)	
2	146 (21.5)	122 (21.9)	24 (19.5)	
≥3	91 (13.4)	73 (13.1)	18 (14.6)	
Diabetes, *n* (%)	213 (31.4)	183 (32.9)	30 (24.4)	0.065
Hypertension, *n* (%)	448 (66.0)	358 (64.4)	90 (73.2)	0.063
Heart disease, *n* (%)	233 (34.3)	198 (35.6)	35 (28.5)	0.13
Living status, *n* (%)				0.009
Accompanied	592 (87.2)	476 (85.6)	116 (94.3)	
Living alone	87 (12.8)	80 (14.4)	7 (5.7)	
Housebound, *n* (%)	126 (18.6)	86 (15.5)	40 (32.5)	<0.001
Albumin, g/L, mean ± SD	40.79 ± 3.21	40.98 ± 3.13	39.91 ± 3.44	<0.001
PHQ-9, median (IQR)	0.0 (0.0, 2.0)	0.0 (0.0, 1.0)	1.0 (0.0, 3.0)	<0.001
PHQ-9 category, *n* (%)				0.002
<5	625 (92.0)	520 (93.5)	105 (85.4)	
≥5	54 (8.0)	36 (6.5)	18 (14.6)	
Falling times in the past year, *n* (%)				<0.001
0	285 (42.0)	263 (47.3)	22 (17.9)	
1	310 (45.7)	237 (42.6)	73 (59.3)	
≥2	84 (12.4)	56 (10.1)	28 (22.8)	
Muscle parameters				
G.Med/MinM area, cm^2^, mean ± SD	32.23 ± 8.24	32.61 ± 8.36	30.55 ± 7.51	0.012
G.Med/MinM density, Hu, mean ± SD	37.54 ± 8.23	38.12 ± 8.09	34.94 ± 8.38	< 0.001
G.MaxM area, cm^2^, mean ± SD	31.48 ± 8.07	31.75 ± 8.25	30.22 ± 7.11	0.057
G.MaxM density, Hu, mean ± SD	30.95 ± 10.30	31.43 ± 10.27	28.77 ± 10.17	0.010

Abbreviations: BMI, body mass index; CCI, Charlson’s comorbidity index; PHQ-9, patient health questionnaire-9; G.MaxM, gluteus maximus muscle; G.Med/MinM, gluteus medius and minimus muscle

### Relationship between muscle parameters and CI

In the unadjusted model, muscle densities were all significantly negatively associated with the risk of CI (*P* < 0.05) as shown in Table 2. Moreover, the G.Med/Min muscle density continued to show a significant association with CI after adjusting for various covariates, with an odds ratio (OR) of 0.77 (95% confidence interval [CI] = 0.62-0.96, *P* = 0.02) (Table 2). Similarly, the density of the G.Max muscle demonstrated a protective trend towards CI after adjustments, although it was not as strong (OR = 0.86, 95%CI = 0.69-1.07). Compared to muscle density, the association of muscle area with CI was notably weaker (Table 2).

**Table 2:** ORs of CI risk per SD increase in sex-specific muscle parameters.

Muscle parameters	CI vs non-CI (123 vs 556)					
	Unadjusted		Model 1^a^		Model 2^b^	
	OR (95% CI)	*P* value	OR (95% CI)	*P* value	OR (95% CI)	*P* value
Muscle density, Hu						
G.Med/MinM density	0.68 (0.56-0.83)	<0.001	0.75 (0.61-0.91)	0.005	0.77 (0.62-0.96)	0.02
G.MaxM density	0.78 (0.64-0.95)	0.012	0.84 (0.68-1.03)	0.088	0.86 (0.69-1.07)	0.177
Muscle area, cm^**2**^						
G.Med/MinM area	0.76 (0.62-0.94)	0.012	0.86 (0.69-1.07)	0.171	0.91 (0.72-1.15)	0.424
G.MaxM area	0.84 (0.68-1.02)	0.084	0.98 (0.78-1.24)	0.894	1.06 (0.83-1.35)	0.641

Abbreviations: CI, cognitive impairment; G.MaxM, gluteus maximus muscle; G.Med/MinM, gluteus medius and minimus muscle.

a Model 1, adjusted for age, sex, and body mass index.

b Model 2, adjusted for Model 1 + CCI, diabetes, education, living status, housebound, albumin, and PHQ-9.

### Sensitivity analysis

The PS distributions of the CI and non-CI groups were highly heterogeneous in the unmatched cohort while balanced after PS matching (Supplementary Figure S1A and B). Nearly all of the SMD values in the PS matching and SMRW cohorts were <0.1 and were far less than those in the unmatched cohort (Supplementary Figure S1C).

**Figure 3:**
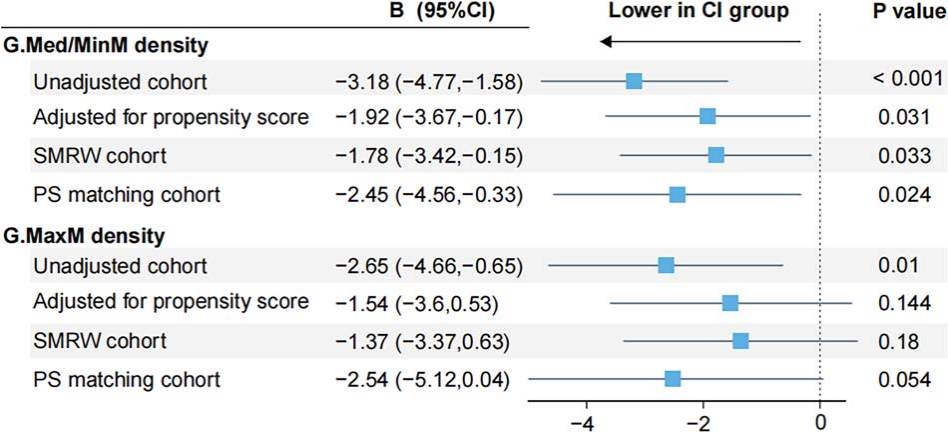
Sensitivity analysis of association between gluteal muscle density and CI based on PS method. Abbreviations: G.Med/MinM, gluteus medius and minimus muscle; G.MaxM, gluteus maximus muscle; CI, cognitive impairment; SMRW, standardized mortality ratio weighting; PS, propensity score.

After adjustments for PS, a significant between-group difference of G.Med/Min muscle density was still found (*B* = –1.92, 95%CI = –3.67--0.17, *P* = 0.031) (Figure 3). Consistent with it, SMRW and PS matched cohorts also revealed significantly lower G.Med/Min muscle density in the CI group than non-CI group (*B* = –1.78, 95%CI = –3.42--0.15, *P* = 0.033; *B* = –2.45, 95%CI = –4.56--0.33, *P* = 0.024, respectively) (Figure 3). Subgroup analysis illustrated the same trend in most subpopulations (Supplementary Figure S2).

## Discussion

Cognitive impairment and muscle weakness are both risk factors for hip fracture in the older adults, posing a serious concern.^9^^,^^10^ These two factors have a complex interplay.^5^ To our knowledge, this is the first study exploring the relationship of muscle parameters derived from CT imaging with cognitive performance in an older population with hip fractures. We targeted muscle size and density through CT measurements and found a significant trend that higher muscle parameters were linked with lower risk for CI. Muscle density displayed a stronger association with cognitive performance than muscle size, especially the G.Med/Min muscle density. In the CI group, the density of the G.Med/Min muscle was approximately 10% lower compared to the non-CI group, with this significant disparity maintaining its robustness in the sensitivity analysis.

Our findings were consistent with most previous muscle imaging studies. The regulation and performance of skeletal muscle are highly relevant to cognitive function. Tessier^7^ followed up with more than eight thousand community-dwelling older adults for 3 years, finding that low muscle mass could suggest accelerated cognitive function decline. Numerous cross-sectional studies have found that sarcopenia defined by either the Asian Working Group for Sarcopenia (AWGS) or the European Working Group on Sarcopenia in Older Person (EWGSOP) were associated with CI.^6^^,^^23^ Chen et al.^24^ further performed a meta-analysis on 26 studies and concluded that patients with sarcopenia had a 1.75 times higher risk for CI than those without sarcopenia. However, few studies have employed CT scanning to obtain accurate muscle characteristics, preventing direct comparisons with our results.

The presented study made a noteworthy distinction between muscle quality and quantity, revealing a stronger correlation between muscle density and CI than muscle size. Muscle density, as measured by the Hounsfield value on CT images, reflects the extent of intramuscular fat infiltration,^12^ better characterizing muscle quality as EWGSOP described.^25^ Compared to muscle size, muscle density exhibited a stronger association with muscle strength and postural balance.^16^^,^^26^ Interestingly, muscle quality, grip strength, and physical performance were also found to be linked to cognitive function.^27–29^ Furthermore, recent evidence suggests that muscle strength and physical fitness are better indicators of CI than muscle mass,^30^^,^^31^ and reciprocally, CI may negatively impact muscle function but not the mass itself.^32^ Indeed, increased focus has been placed on the importance of quality over quantity in the past decade. Most international consensus statements have now emphasized muscle function more than muscle mass.^25^^,^^33^ Nevertheless, still, the operational definition of muscle quality remains controversial. In the future, it is expected that CT or magnetic resonance imaging may assume a more prominent role in assessing quality due to its unique advantage in tissue differentiation.

Adipose tissue located within muscle has been identified as an endocrine organ that regulates the muscular environment. Adipokines and lipokines, which are signaling molecules derived from adipose tissue, are involved in glycometabolism and inflammation in skeletal muscle..^34^ Therefore, disruptions in muscle metabolism could be correlated with insulin resistance and fluctuations in inflammatory factors. A recent animal study found that muscle insulin resistance can lead to reduced hippocampal neurogenesis, which may contribute to cognitive decline..^35^ These findings could partly explain the results of our study and indicate an underlying association between muscle density and insulin resistance, which warrants further investigation. In addition, exercise-induced myokines have been shown to have protective effects against CI and may increase levels of brain-derived neurotrophic factors.^5^ However, caution should be taken until more reliable causal evidence is available regarding the effects of physical exercise on cognitive enhancement.^36^

Furthermore, our study has uncovered that G.Med/Min muscle density is significantly associated with CI, even after adjusting for confounding factors. The G.Med/Min muscle, the dominant abductor for the hip, plays a crucial role in maintaining balance during standing and walking.^37^ In contrast to the G.Max muscle, which is mostly quiescent with low levels of activity, G.Med/Min muscle takes the primary responsibility for daily activities in the older adults.^38^ Previous prospective cohort studies have identified G.Med/Min muscle density as an independent risk factor for both the first and second hip fractures.^13^^,^^14^ Our findings suggest that this muscle may also be a potential target for future interventions aimed at preventing or treating cognitive decline.

The notable strengths of our study are that, firstly, we carefully controlled for key confounding factors, including education, living alone, housebound status, albumin level, depression disorder, and baseline medical conditions, which reduce the likelihood of alternative explanations for the observed associations. Secondly, we employed PS methods as sensitivity analyses to ensure the robustness of the association between G.Med/Min muscle density and CI, enhancing the reliability of our findings. Thirdly, we minimized the potential impact of fracture-related changes on hip muscle by selecting patients who underwent CT scans within 72 hours of their hip fracture.

This study has a few limitations. First, The cross-sectional design prevents us from establishing causality, allowing us only to identify associations. Second, our research focused exclusively on patients with hip fractures, assessing muscle density on the contralateral, non-fractured side after excluding individuals with pre-existing mobility problems. Consequently, the applicability of our findings to broader populations is restricted. Third, the collection of data on potential confounders, such as medication usage and exercise habits, was insufficient. These aspects offer opportunities for more rigorous control in future research.

In conclusion, our investigation has revealed a significant correlation between increased muscle parameters and a reduced risk of CI. Notably, muscle density, particularly in the G.Med/MinM muscle, exhibited a stronger association with cognitive performance than muscle size. These findings suggest an important interplay between skeletal muscle and cognitive function, indicating that muscle density may be a promising candidate for future studies on cognition.

## Supplementary Material

Supplement_material_ziae047

## Data Availability

The datasets used in this study are not publicly available because of participant confidentiality but are available from the corresponding author on reasonable request.
