# Supplementary material for: Muscle density, but not size, is independently associated with cognitive health in older adults with hip fractures
Source: JBMR Plus. 2024 Apr 2;8(5):ziae047. doi: 10.1093/jbmrpl/ziae047 (PMC11044827; doi:10.1093/jbmrpl/ziae047)
Supplement: Supplement_material_ziae047 [file supplement_material_ziae047.docx]

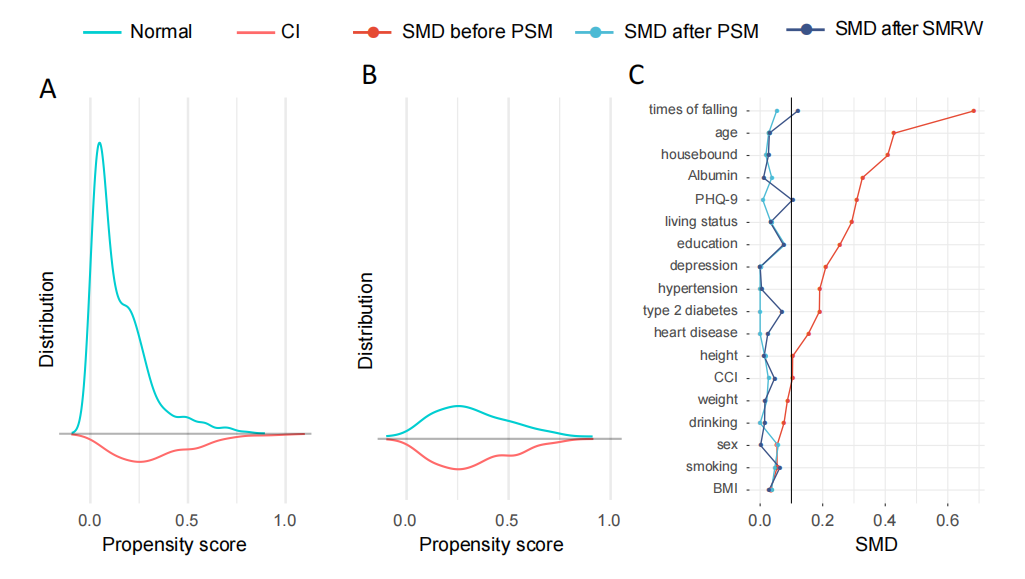


**Supplementary Fig.S1** Propensity score distribution and SMD of various cohorts.

(A) unmatched cohort; (B) propensity score matched cohort; (C) the standardized mean difference of the variables in the unmatched, PSM, and SMRW cohorts

Abbreviation: CI, cognitive impairment; BMI, bone mass index; CCI, Charlson’s comorbidity index; PHQ-9, patient health questionnaire-9; PSM, propensity score matching; SMRW, standardized mortality ratio weighting; SMD, standardized mean difference;


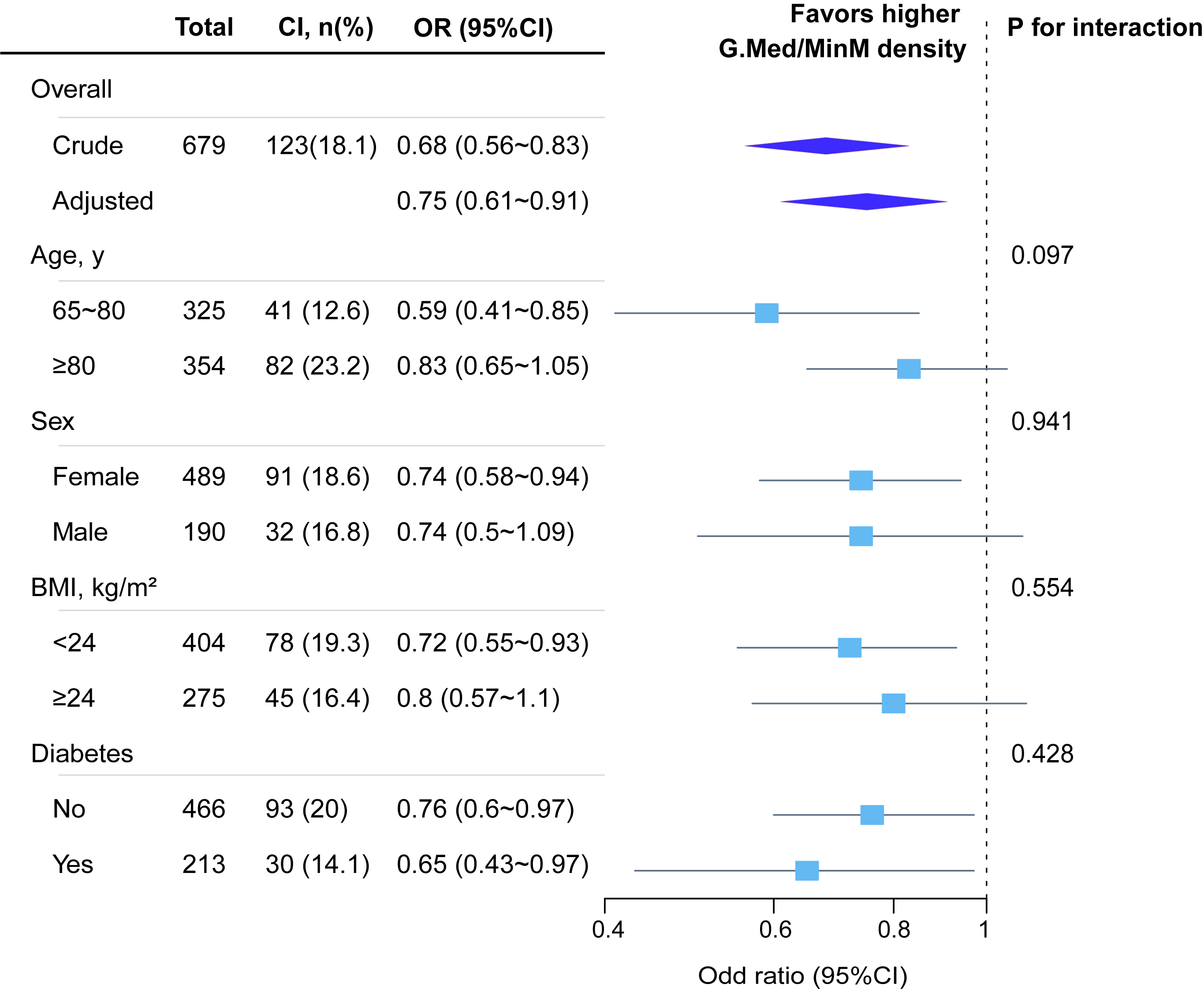


**Supplementary Fig.S2** ORs of CI risk per SD increase in sex-specific G.Med/Min muscle density in different subgroups

Abbreviation: G.Med/MinM, gluteus medius and minimus muscle; CI, cognitive impairment; BMI, bone mass index;
